# Glucagon‐like peptide‐1 and glucose‐dependent insulinotropic peptide: effects alone and in combination on insulin secretion and glucose disappearance in mice

**DOI:** 10.14814/phy2.13280

**Published:** 2017-06-14

**Authors:** Giovanni Pacini, Bo Ahrén

**Affiliations:** ^1^ Metabolic UnitInstitute of Neuroscience (IN‐CNR)PadovaItaly; ^2^ Department of Clinical Sciences LundLund UniversityLundSweden

**Keywords:** Beta cell function, glucose effectiveness, incretin hormones, insulin sensitivity, intravenous glucose test, mathematical modeling

## Abstract

Glucagon‐like peptide‐1 (GLP‐1) and glucose‐dependent insulinotropic peptide (GIP) stimulate insulin secretion. They are both released after meal ingestion, and therefore they might cooperate in their actions. However, whether there is a cooperative action of the two incretins is not known. This study therefore investigated the effects on insulin secretion and glucose disappearance of GLP‐1 and GIP when given together with intravenous glucose both alone and in combination in mice. Four different doses were used (0.003, 0.03, 0.3 and 3.0 nmol/kg), which ranged from subthreshold to maximal doses to stimulate first‐phase insulin secretion as evident from initial dose–response studies. It was found that at 0.03 nmol/kg and higher doses, glucose‐stimulated insulin secretion was augmented by both incretins. When they were given in combination, no further increase was observed, indicating no synergistic effect. Also, glucose disappearance rate was increased by 0.03 and 3.0 nmol/kg of the two incretins, both when they were given alone and in combination with, again, no synergy. Finally, glucose effectiveness (an index of noninsulin‐mediated processes) was enhanced by the two incretins, in particular GIP. We also found that insulin‐dependent and insulin‐independent mechanisms contributed 38% and 62%, respectively, to glucose tolerance after glucose alone; with GIP, the contribution by noninsulin‐dependent processes was remarkably dominant and with GLP‐1, insulin‐dependent processes were prevailing. In conclusion, GIP and GLP‐1 stimulate insulin secretion and glucose effectiveness in mice with no synergistic action, but with a dissociated contributory effector on glucose disposal: with GLP‐1 being more active on insulin‐dependent processes and GIP more active on noninsulin‐dependent processes.

## Introduction

Glucagon‐like peptide‐1 (GLP‐1) and glucose‐dependent insulinotropic peptide (GIP) are both incretin hormones which are released following meal ingestion (Kreymann et al. 1987; Elliott et al. 1993; Nauck et al. 1993; Carr et al. 2010). They contribute to a large extent to insulin secretion during a meal (Nauck et al. 1993) and, as has been reviewed, they are of relevance for physiology and pathophysiology of type 2 diabetes (Deacon and Ahrén 2011; Drucker 2013). GLP‐1 and GIP stimulate insulin secretion through direct effects on beta cells by activating specific receptors and they share several mechanistic signals (Thorens 1992; Gremlich et al. 1995). GLP‐1 has also been shown to stimulate insulin secretion through a neural mechanism (Ahrén 2014). In model experiments in mice, we have compared their effects and found that GLP‐1 mainly accelerates the onset of insulin secretion, making the first phase particularly prominent (Thomaseth et al. 2007). Furthermore, GIP is more efficient in augmenting second phase of insulin release (Pacini et al. 2010). These effects are important for the understanding of the function of the incretin hormones, but equally important is to explore whether the two incretins cooperate in stimulating insulin secretion. A cooperation between the two incretins is relevant since they are both released after food ingestion (Carr et al. 2010), and since their circulating levels are both increased during treatment with dipeptidyl peptidase‐4 (DPP‐4) inhibition, when the inactivation of both GLP‐1 and GIP is prevented (Carr et al. 2010). However, whether the two incretins indeed cooperate by having additive or synergistic effects to stimulate beta cell function is not known. The rationale for the present study, therefore, was to explore the effects of submaximal doses of GLP‐1 and GIP both alone and in combination on insulin secretion and glucose disappearance in mice.

## Materials and Methods

### Animals and experimental protocols

Experiments were performed in female C57BL/6 mice (Taconic, Skensved, Denmark). Animals were 4 weeks old on arrival. After 8 weeks of feeding with a standard rodent diet with 10% of energy from fat (D12450B, Research Diets, New Brunswick, NJ), yielding a body weight of 25 ± 3(SD) g, at the day of the experiment, the mice were anesthetized with an intraperitoneal injection of midazolam (0.4 mg/mouse or 18 mg/kg, Dormicum; Hoffman‐La Roche, Basel, Switzerland) and a combination of fluanisone (0.9 mg/mouse) and fentanyl (0.02 mg/mouse or 41/9 mg/kg, Hypnorm; Janssen, Beerse, Belgium). The intravenous glucose tolerance test (IVGTT) experiment was performed as follows: a basal blood sample was taken from the retrobulbar, intraorbital, capillary plexus in heparinized tubes. Then, mice were injected intravenously in a tail vein (total volume load 10 *μ*L/g of body weight) with either glucose alone (0.35 g/kg; Sigma‐Aldrich, St. Louis, MO) or together with synthetic porcine GIP (Bachem, Bubendorf, Switzerland) and/or synthetic human GLP‐1 (Sigma‐Aldrich) at each of three different dose levels (0.003, 0.03, and 3.0 nmol/kg, called subthreshold, submaximal, and maximal doses, respectively). An additional dose of 0.3 nmol/kg was also included in a dose–response study. In all individual experiments, groups of mice with glucose alone and glucose with GIP and/or GLP‐1 were included for a given dose of hormone. This means that all experiments were controlled to test the question of GIP/GLP‐1 interactions at the three doses. This study design thus enables that all comparisons are validly controlled and the design also implies that the total number of animals with a glucose control is higher than the other experimental groups. Table 1 shows the number of animals in each group. Samples after the injection (40 *μ*L each) were taken at 1, 5, 10, 20, and 50 min from the retrobulbar plexus. The study was approved by the regional ethics committee in Lund, Sweden.

**Table 1:** Number of animals in each individual experimental group

Glucose alone (0.35 g/kg)	106
Glucose + GIP (0.003 nmol/kg)	12
Glucose + GLP‐1 (0.003 nmol/kg)	11
Glucose + GIP + GLP‐1 (0.003 nmol/kg)	12
Glucose + GIP (0.03 nmol/kg)	28
Glucose + GLP‐1 (0.03 nmol/kg)	25
Glucose + GIP + GLP‐1 (0.03 nmol/kg)	15
Glucose + GIP (0.3 nmol/kg)	28
Glucose + GLP‐1 (0.3 nmol/kg)	25
Glucose + GIP (3.0 nmol/kg)	40
Glucose + GLP‐1 (3.0 nmol/kg)	47
Glucose + GIP + GLP‐1 (3.0 nmol/kg)	29

### Assays

Plasma samples were immediately obtained by separation with centrifugation and stored at −20°C until analysis. Insulin was measured by ELISA (Mercodia, Uppsala, Sweden). The intra‐assay coefficient of variation of the method is 4% at both low and high levels, while the interassay coefficient of variation is 5% at both low and high levels. The lower limit of quantification of the assay is 6 pmol/L. Plasma glucose concentrations were determined using the glucose oxidase method.

### Data analysis

Since it is difficult to distinguish between first‐ and second‐phase insulin release during an IVGTT, we considered the insulin peak (concentration at 1 min sample) as representative of the early‐phase insulin response. The total area under the insulin curve (AUC_insulin_) describes the total insulin release. Incremental AUC (ΔAUC) is the area over the basal level. Beta cell function was evaluated as AUC_insulin_ divided by AUC_glucose_.

Glucose disappearance rate was estimated as the net glucose elimination rate after the glucose injection: *K*_G_, the glucose tolerance index. It was calculated as the slope for the 5–20 min interval after glucose injection of the logarithmic transformation of the individual plasma glucose values (Pacini et al. 2009). Insulin sensitivity and glucose effectiveness were evaluated with the minimal model technique as extensively explained elsewhere (Ahrén and Pacini 1999; Pacini et al. 2001, 2009, 2013). The method provides parameters *S*_I_ (insulin sensitivity index), defined as the ability of insulin to enhance net glucose disappearance and inhibit glucose production, and *S*_G_ (glucose effectiveness) which represents net glucose disappearance per se from plasma without any change in dynamic insulin. AUCs were calculated with the trapezoidal rule. Assessment of contribution by insulin‐dependent and insulin‐independent mechanisms to glucose tolerance was performed by using *S*_G_ and AUC_insulin_ in the multiple regression with *K*_G_, as described previously extensively (Pacini et al. 2001). Comparisons were performed with Student's *t‐*test. Data and results are shown as mean ± SE, unless otherwise designated.

## Results

### Insulin secretion

**Figure 1:**
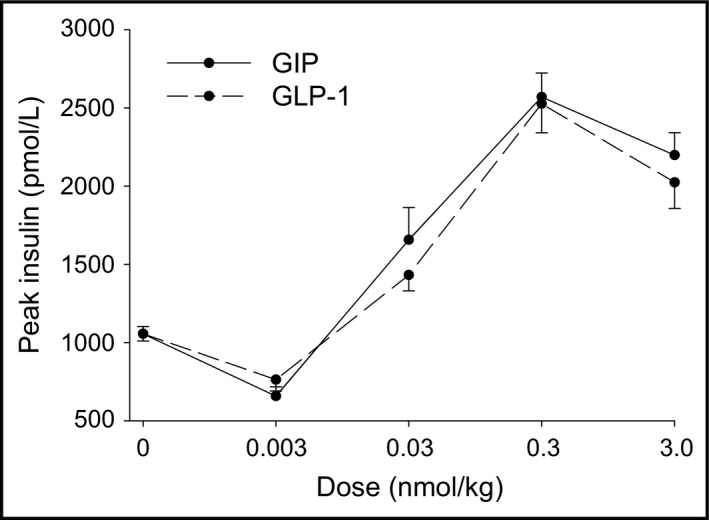
Peak (1 min) insulin (mean ± SE) after intravenous injection of glucose (0.35 g/kg) alone or with either GIP or GLP‐1 at 0.003, 0.03, 0.3, or 3.0 nmol/kg. Number of animals in each group is reported in Table 1.

**Figure 2:**
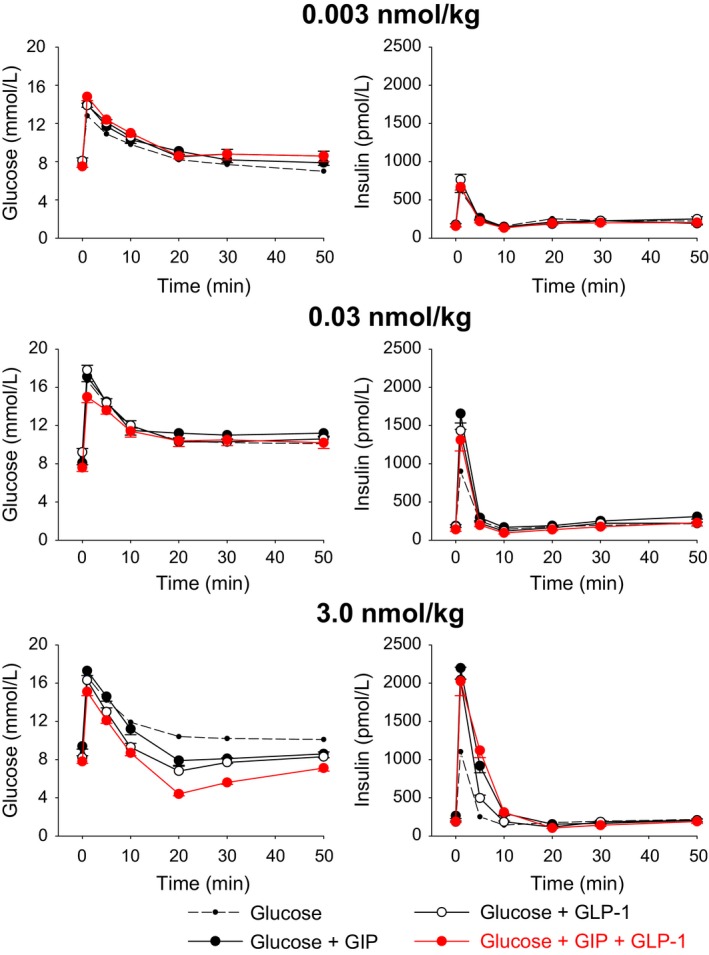
Glucose and insulin concentration time courses (mean ± SE) after intravenous injection of glucose (0.35 g/kg) alone or with either GIP or GLP‐1 at 0.003 nmol/kg (subthreshold dose), 0.03 nmol/kg (submaximal dose), or 3.0 nmol/kg (maximal dose) or with the two incretin hormones in combination. Number of animals in each group is reported in Table 1.

Figure 1 shows first‐phase insulin secretion (insulin peak at 1 min) for glucose alone and for GIP and GLP‐1 in combination with glucose at 0.003, 0.03, 0.3, and 3.0 nmol/kg. It is seen that there was a clear dose‐dependent effect to potentiate insulin secretion for the two incretin hormones. It is also evident that at the dose of 0.003 nmol/kg, no augmentation of the insulin response to glucose was evident by GIP or GLP‐1 (i.e., at a subthreshold dose), and that 0.3 and 3.0 nmol/kg are maximal doses. For the combination studies, 0.003, 0.03, and 0.3 nmol/kg were selected and studies were undertaken with GIP and GLP‐1 alone and in combination at these dose levels. Figure 2 shows the time courses of insulin and glucose at these dose levels. GIP and GLP‐1 both augmented the insulin peaks compared to glucose alone at the 0.03 and 3.0 nmol/kg doses, and insulin levels (both peak levels and AUC) were also higher after injection of the incretins in combination with glucose compared to glucose alone throughout the 50‐min study period (Table 2). However, when given in combination, GIP+GLP‐1 yielded insulin levels that were not different from those reached with the two peptides given alone at any of these doses (Table 2 and Fig. 2).

**Table 2:** Insulin peak concentration (at 1 min), suprabasal AUC for insulin and glucose concentrations, beta cell function (BCF) and measures derived from the IVGTT, plus percent contribution to glucose disappearance of insulin‐independent (*S*_G_) and insulin‐dependent (AUC_insulin_) processes, when GLP‐1 and GIP are given together with glucose at 3.0 nmol/kg

	Glucose only (0.35 g/kg)	Glucose + Incretin hormones at maximal dose (3.0 nmol/kg)		
		GIP	GLP‐1	GIP + GLP‐1
Peak insulin (pmol/L)	1056 ± 46	2198 ± 144^b^	2042 ± 167^b^	2024 ± 188^b^
ΔAUC_insulin_ (min·nmol/L)	3.85 ± 0.41	11.43 ± 1.11^b^	8.39 ± 0.98	11.06 ± 1.51^b^
AUC_glucose_ (min·mol/L)	555 ± 11	455 ± 17^b^	474 ± 16^a^	423 ± 18^b^
BCF (mmol_insulin_/mol_glucose_)	0.022 ± 0.001	0.048 ± 0.003^b^	0.037 ± 0.004	0.049 ± 0.005^b^
Glucose tolerance, *K*_G_ (%min^−1^)	2.05 ± 0.09	4.76 ± 0.32^b^	4.43 ± 0.33^a^	5.25 ± 0.45^b^
Insulin sensitivity, *S*_I_ (10^−4^ min^−1^/[pmol/L])	1.10 ± 0.06	0.92 ± 0.05	1.21 ± 0.08	1.11 ± 0.08
Glucose effectiveness, *S*_G_ (min^−1^)	0.045 ± 0.003	0.072 ± 0.004^b^	0.066 ± 0.005^a^	0.082 ± 0.007^b^
Percent contribution to glucose disappearance of processes (±coefficient of variation, as SD)				
Insulin independent (*S*_G_)	61.3 ± 1.6	75.8 ± 6.4	34.6 ± 1.8	38.2 ± 6.1
Insulin dependent (AUC_insulin_)	38.7 ± 1.6	24.2 ± 6‐4	65.4 ± 1.8	61.8 ± 6.1

a *P*‐value range between 0.0004 and 0.040.

b *P* < 0.0001 (both for comparison with the corresponding values of glucose only).

Since glucose levels were reduced in the experiments with the incretin hormones at the 3.0 nmol/kg dose, their influences on beta cell function were estimated by insulin levels in relation to glucose at this dose (Table 2). It was thereby shown that beta cell function was similarly increased by adding GIP singularly to glucose at submaximal doses compared with glucose alone. Also, GLP‐1 slightly increased beta cell function, though not significantly. No additional effect was observed when the two incretins were given together (Table 2).

### Glucose disappearance

Also, glucose levels peaked at 1 min. The subsequent glucose elimination was not affected by the administration of the incretin hormones at 0.003 and 0.03 nmol/kg, either alone or in combination as shown in Figure 2. In contrast, at 3.0 nmol/kg, glucose elimination rate was enhanced both with GIP and GLP‐1 given alone and in combination, with no significant difference between the groups (Fig. 2, and *K*_G_ shown in Table 2). However, with the combination, the 20‐min glucose level was lower than with either of the two incretin hormones alone (4.4 ± 0.2 mmol/L vs. 7.9 ± 0.5 mmol/L with GIP, 6.8 ± 0.5 mmol/L with GLP‐1, and 10.4 ± 0.2 mmol/L for glucose alone; *P* < 0.001 for the combination vs. the GIP and GLP‐1, respectively). Parameters related to glucose kinetics are reported in Table 2 for the 3.0 nmol/kg dose. Insulin sensitivity did not exhibit appreciable changes by the incretin hormones, alone or in combination, when compared to glucose only at any dose (not shown). Glucose effectiveness (*S*_G_) was instead markedly increased, although there was no difference between the incretin hormones in combination compared to GIP or GLP‐1 alone. There was no association between *S*_G_ and AUC_insulin_ after i.v. injection of glucose alone and of glucose with GIP (no significant regression: *P* = 0.296 and *P* = 0.951, respectively). In contrast, after GLP‐1 there was a weak, but significant correlation between *S*_G_ and AUC_insulin_ (*r* ^2^=0.136, *P* = 0.01); the combination of the incretins yielded no association (*P* = 0.12).

### Relative contribution of insulin‐dependent versus ‐independent processes

Table 2 shows also the percent contribution to glucose disappearance of insulin‐dependent versus insulin‐independent mechanisms for the various groups at 3.0 nmol/kg. In the control animals, 61% of intravenous net glucose disappearance was due to *S*_G_ and 39% due to AUC_insulin_. GIP increased the relative contribution by *S*_G_, whereas GLP‐1 increased the relative contribution by AUC_insulin_. The combined group had a similar contribution of the two mechanisms as GLP‐1 alone.

## Discussion

This study explored the potential cooperation between the two incretin hormones GIP and GLP‐1 on first‐phase insulin secretion and glucose elimination in mice. The rationale for the study was that circulating levels of both incretins are raised following meal ingestion and, therefore, under physiological conditions they may work in concert (Carr et al. 2010). Furthermore, also during treatment with DPP‐4 inhibitors, the levels of both GIP and GLP‐1 (active forms) are increased (Carr et al. 2010) and thus they may be working in cooperation also under this condition. We employed a model in which the two hormones were administered individually or together in combination with glucose to study insulin secretion and glucose disappearance under in vivo conditions. Furthermore, we used several doses of the hormones, ranging from subthreshold to maximal doses of the hormones. Since the insulin response to intravenous glucose is short lived and mainly related to first‐phase insulin secretion, our conclusion is mainly restricted to combination effects on first‐phase insulin secretion.

The results show that GIP and GLP‐1 both stimulate insulin secretion and augment glucose elimination. Furthermore, when comparing the dose–response relationship between dose of GIP or GLP‐1 and insulin secretion, it was evident that the two incretin hormones are equipotent in stimulating first‐phase insulin secretion over a wide dose range. However, a main finding of this study is that they do not exhibit any cooperative effect on these processes. Instead, the effects of the combination of GIP plus GLP‐1 are not different from the effects of either hormone alone. This shows that there is no additional effect on insulin secretion when both incretins are present together compared to each of them alone, and this is evident at different dose levels, ranging from subthreshold to maximal levels. Our data at low doses are different from the results by Nauck et al. (1993), who showed that combination of physiological doses of GIP and GLP‐1 are additive when tested in a hyperglycemic clamp study in humans. This might suggest a potential species difference, but on the other hand, it is important to emphasize that our present conclusion is restricted to the first (early)‐phase insulin secretion, whereas a study in hyperglycemic clamp conditions is more focused on the second (late)‐phase insulin secretion. Therefore, further studies focusing explicitly on the second‐phase insulin secretion are therefore warranted in mice. Interestingly, our results are similar to our previous observation that there is no additional effect when both GLP‐1 and glucagon are given together during an IVGTT (Pacini and Ahrén 2015).

Both GIP and GLP‐1 potently exaggerated glucose elimination at 3.0 nmol/kg, as evident from the reduced AUC_glucose_ and the increased *K*_G_, the latter being more than twice as much than with glucose alone. The analysis of the relationship between glucose disappearance and insulin concentration revealed that insulin sensitivity did not change by the two incretin hormones. This was expected with GLP‐1, as we already demonstrated (Ahrén and Pacini 1999); instead, the new finding is that also the acute administration of GIP has no effect on insulin sensitivity and, again, the combination of the two incretins did not change the effect of the two hormones on insulin sensitivity.

It is worth noting that at the dose of 3.0 nmol/kg, the glucose nadir was lower after the GIP + GLP‐1 combination than after each incretin hormone alone. Since insulin levels were not significantly higher after the combination versus the individual hormones, this may suggest an insulin‐independent increase in glucose elimination by the combination. Glucose levels, however, never reached hypoglycemia, which may be due to a robust glucose counter‐regulation, including glucagon counter‐response which is evident for the incretin hormones, in particular GIP (Malmgren and Ahrén 2015). Nevertheless, this strong glucose reducing effect of the combination of GIP and GLP‐1 deserves to be examined further from a point of view of insulin‐independent mechanisms. In fact, another stimulating issue of this study is the possible relationship between the changes in the incretin hormones and the increased glucose effectiveness (*S*_G_). This parameter reflects the action of hyperglycemia independent on elevated insulin in stimulating glucose uptake and suppressing endogenous glucose production (Ader et al. 1997). The ability of hyperglycemia to accelerate glucose uptake even in the absence of a sustained pancreatic insulin response was noted in humans from the dose–response relationship between insulin and glucose uptake (Best et al. 1981) and extensively studied in dogs (Ader et al. 1985). The increase in noninsulin‐mediated glucose disappearance with elevated incretins was already observed with IVGTT more than 20 years ago (D'Alessio et al. 1994). Our results show that GLP‐1 augments *S*_G_ by 31.8% (Table 2), very consistent with what found in that study in humans where a 30.8% increase was observed (D'Alessio et al. 1994). Moreover, it has been shown that during the early phase of the oral glucose tolerance test (OGTT), glucose uptake is dominated by glucose effectiveness (Best et al. 1996). This study was carried out when the concept of incretins was not yet fully introduced, therefore no mention to them was made. Nonetheless, it is relevant to point out that in the early phase of OGTT the concentrations of both GIP and GLP‐1 are elevated. The results in mice of the present study show that in the presence of high GIP and GLP‐1 the action of hyperglycemia (insulin‐independent) on glucose clearance is stimulated, as shown by the increased *S*_G_, confirming that found in humans (Vahl et al. 2003).

The reason why glucose effectiveness is increased by the incretins is not clear. To the best of our knowledge, no ad hoc studies have been performed to understand the biochemical pathways or the (molecular) biology mechanisms involved in the processes responsible of this behavior. Our in vivo model in the whole animal cannot provide further information. Studies in dogs (Ader et al. 1997) and humans (Saccà et al. 1978) indicate the importance of the suppression of hepatic glucose production by hyperglycemia per se, which is quantified by *S*_G_. A possible involvement of free fatty acid metabolism has also been suggested (Best et al. 1996). The main question remains as to whether the increase in glucose effectiveness is due either to the increasing insulin, regardless of the stimulus, or to a primary effect of incretins. Evidences from other studies seem to yield a propensity for the first hypothesis, being a similar increase of *S*_G_ observed in another mice strain (NMRI) when the animals were injected with glucose plus one of three different insulin stimulatory compounds, namely GLP‐1, synthetic ovine pituitary adenylate cyclase‐activating polypeptide, and synthetic COOH‐terminal octapeptide of cholecystokinin (Pacini et al. 2001). In addition, a sustained augmentation of *S*_G_ has been observed in these animals also when injected with increasing doses of insulin together with glucose: that is, without any addition of GLP‐1 or other secretagogues (Pacini et al. 2001). Finally, this fact was also revealed in humans with IVGTT (thus, no increase in incretins), where it was observed that subjects undergoing both regular and insulin‐modified IVGTT exhibited in the second test a systematically elevated *S*_G_ that correlated with the increased insulin (Pacini et al. 1998). Therefore, from these evidences, it appears that the incretins seem to have effect on glucose effectiveness via an indirect effect through elevating insulin concentration. However, in the present study, carried out in a large number of animals, no association between *S*_G_ and AUC_insulin_ was observed; thus, we cannot exclude in our mice the presence of a primary direct effect on the disappearance of glucose per se, especially as concerns GIP. This would be consistent with findings of extrapancreatic actions of GIP (Hansotia et al. 2007; Seino and Yabe 2013) and, in particular, that GIP could be involved in lipid metabolism in adipocytes by affecting both lipolysis and lipid re‐esterification (Gögebakan et al. 2012; Yamada et al. 2016), which may be indeed related to actions on glucose metabolism. GLP‐1 has been also shown to have extrapancreatic effects; in particular, hepatic effects of GLP‐1 have been demonstrated in suppressing glucose production and therefore these effects are of remarkable importance for glucose disappearance (Seghieri et al. 2013). Moreover, C‐terminal fragments of GLP‐1 have been indicated to have effects in the liver (Tomas et al. 2011); and finally, it has also been observed that GLP‐1 may activate neural circuits (Kakei et al. 2002) in stimulating insulin secretion (Krieger et al. 2016). In keeping with the results of the present study, it would be an interesting hypothesis that such a stimulated circuit has also indirect effects on glucose elimination per se, but this needs to be studied in more detail.

A further aspect that was evaluated in this study is the relative effects on *K*_G_ of AUC_insulin_ and *S*_G_, respectively. It is in fact important to quantify the relative contribution of insulin‐mediated and dynamic noninsulin‐dependent mechanisms on glucose disappearance (*K*_G_) in the various conditions, for a complete understanding of the processes involved in glucose disappearance. In humans, for instance, it has been shown with the glucose clamp that, at basal insulin, the contribution of the noninsulin‐mediated processes accounts for at least 60% of the uptake (Baron et al. 1988), and decreases to 30% at high insulin (Best et al. 1996). We found that following glucose alone, *S*_G_ was to some extent more important than AUC_insulin_ to increase *K*_G_ (61% vs. 39%, Table 2). This finding is, however, slightly different from what was found in previous studies (Pacini et al. 2001), which were done in another mouse strain. In NMRI mice, the percent contribution of *S*_G_ versus insulin was 70% versus 30%, respectively, for similar levels of insulin sensitivity. The new finding here is that the proportion of the relative contribution of insulin‐dependent and noninsulin‐dependent mechanisms for glucose disposal was markedly changed from the glucose alone situation after GIP. Thus, GIP‐induced increase in *K*_G_ was associated with a much higher dependency on *S*_G_ than both after glucose alone and after glucose plus GLP‐1. This would suggest that GIP enhances the processes driving noninsulin‐dependent glucose clearance, which, again, would fit with extrapancreatic actions of GIP (Hansotia et al. 2007). In contrast, after GLP‐1 alone the contribution by insulin‐dependent processes was increased, despite overall less insulin, showing that the insulinotropic effect of GLP‐1 is to be the main mechanism underlying its increase in *K*_G_. These findings demonstrate a dissociation between the two incretins with regard to mechanisms stimulating glucose clearance.

In conclusion, our study shows that GIP and GLP‐1 both stimulate insulin secretion and consequently glucose elimination is augmented in mice. However, even at a submaximal dose, the effects of the combination of GIP plus GLP‐1 are not different from the effects of either hormone alone, indicating no additional effect from the incretins together compared to each of them alone. Both insulin‐mediated and noninsulin‐mediated processes contribute almost equally to glucose disappearance, when glucose is given alone. Glucose disappearance rate markedly increases when incretins are given. When GIP is given alone with glucose, the contribution of the noninsulin‐mediated processes is higher than that with glucose alone, in accordance with possible extrapancreatic effects of GIP. In all cases, when both incretins are given together, the overall behavior is similar to that obtained with GLP‐1 alone, which may suggest that indirect effects may be of more importance for the action of GLP‐1 (Krieger et al. 2016). The results therefore indicate (1) that there is no synergistic effect of the combination of GIP and GLP‐1 in mice, (2) that GLP‐1 overrides the effect of GIP both in terms of insulin secretion and glucose disappearance, and (3) that the two incretins have different relative dependency on insulin‐dependent and noninsulin‐dependent mechanisms for their action to stimulate glucose disposal.

### Perspective and Significance

The incretin hormones GIP and GLP‐1, when given alone intravenously with glucose, yield a sustained hyperinsulinemia; our study highlights the fact that there is no synergistic effect of GLP‐1 and GIP in mice, when given in combination at doses over a wide range, on both insulin secretion and glucose disappearance. This shows that the combined increase in both incretins after meal ingestion is less relevant for peak insulin secretion after a challenge, but rather that the combination may be relevant for other effects. Furthermore, the difference in relative contribution of insulin‐dependent versus noninsulin‐dependent mechanisms for the stimulation of glucose disposal by GLP‐1 and GIP suggests that they have different main mediators for stimulating glucose disposal: GLP‐1 being more dependent on insulin and GIP more dependent on noninsulin, presumably extrapancreatic, effects. This effect of GIP may be relevant for the stimulation of glucose disposal during insulinopenia, which, however, deserves further thorough investigations.

## Conflict of Interest

The authors declare no conflict of interests related to this work.
